# Nondegenerated cystic neuroendocrine tumor of the pancreas: a case report

**DOI:** 10.1186/s40792-020-00918-6

**Published:** 2020-06-29

**Authors:** Keisuke Noda, Tamotsu Kuroki, Mampei Yamashita, Takanori Hirayama, Koji Natsuda, Shinichiro Kobayashi, Takayuki Tokunaga, Kosho Yamanouchi, Hiroaki Takeshita, Shiro Miura, Shigeto Maeda

**Affiliations:** 1 Department of Surgery, National Hospital Organization Nagasaki Medical Center, 2-1001-1, Kubara, Omura City, Nagasaki 856-8562 Japan; 2 Department of Pathology, National Hospital Organization Nagasaki Medical Center, Omura, Japan

**Keywords:** Pancreatic neuroendocrine tumor, Cystic lesion, Pancreas

## Abstract

**Background:**

Pancreatic neuroendocrine tumors (PNETs) are typically solid neoplasms but, in very rare cases, present as cystic lesions. We describe a case of a cystic neuroendocrine tumor that developed as a small cystic lesion.

**Case presentation:**

In 2011, a 66-year-old Japanese woman underwent computed tomography (CT) that revealed a cystic lesion in the tail of the pancreas measuring 9 mm. She did not have any symptoms. She underwent a CT scan every year thereafter. The cystic lesion gradually increased and was 40 mm in 2019; endoscopic retrograde pancreatography (ERP) was then performed. Cytological examination demonstrated class IIIb adenocarcinoma, and we conducted laparoscopic distal pancreatectomy. Pathological examination showed PNET.

**Conclusion:**

Although cystic change of PNET is generally caused by ischemia or necrosis inside the tumor, in our case, PNET occurred as a small cyst that increased without changing form.

## Background

Pancreatic neuroendocrine tumors (PNETs) are very rare, accounting for only 1 to 2% of all pancreatic tumors [1]. PNETs are typically solid neoplasms but present as cystic lesions in very rare cases [2]. In general, the mechanism of cystic degeneration in PNET is ischemia or necrosis inside the tumor because of an increase in tumor diameter or intratumoral hemorrhage due to blood vessel rupture [2].

Herein, we describe a case of a cystic PNET that developed as a small cystic lesion.

## Case presentation

**Fig. 1:**
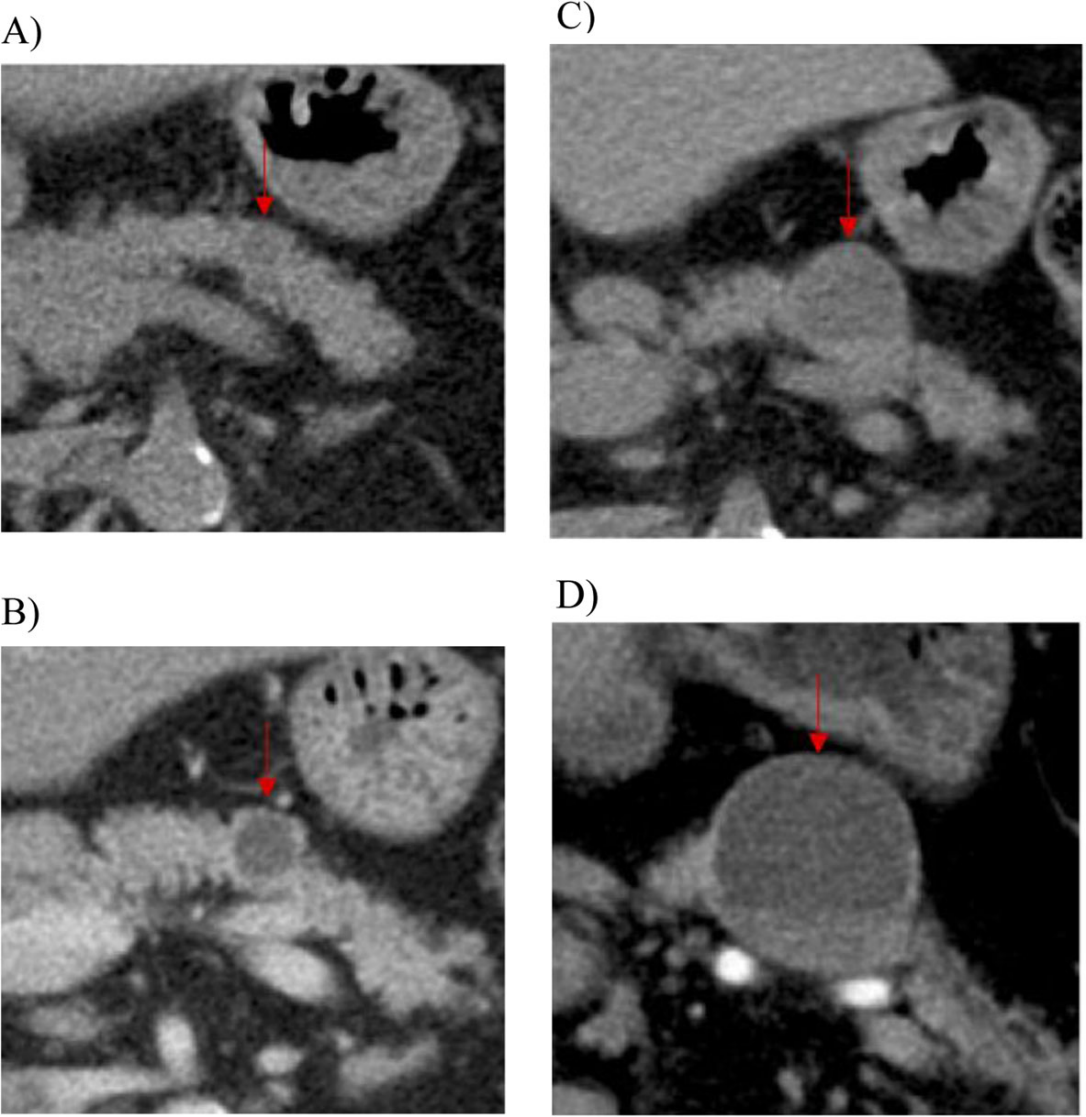
Abdominal CT showing the cystic lesion in the tail of the pancreas that had become larger without a change in form. **a** Unenhanced CT showing a 9-mm cystic lesion in 2011. **b** Contrast-enhanced CT (CECT) showing a 14-mm cystic lesion in 2013. The peripheral rim was enhanced. **c** Unenhanced CT of a 33-mm cystic lesion and consisting of fluid at the bottom, which was considered clot in 2018. **d** CECT showing a 40-mm cystic lesion in 2019. The outer rim became thinner

**Fig. 2:**
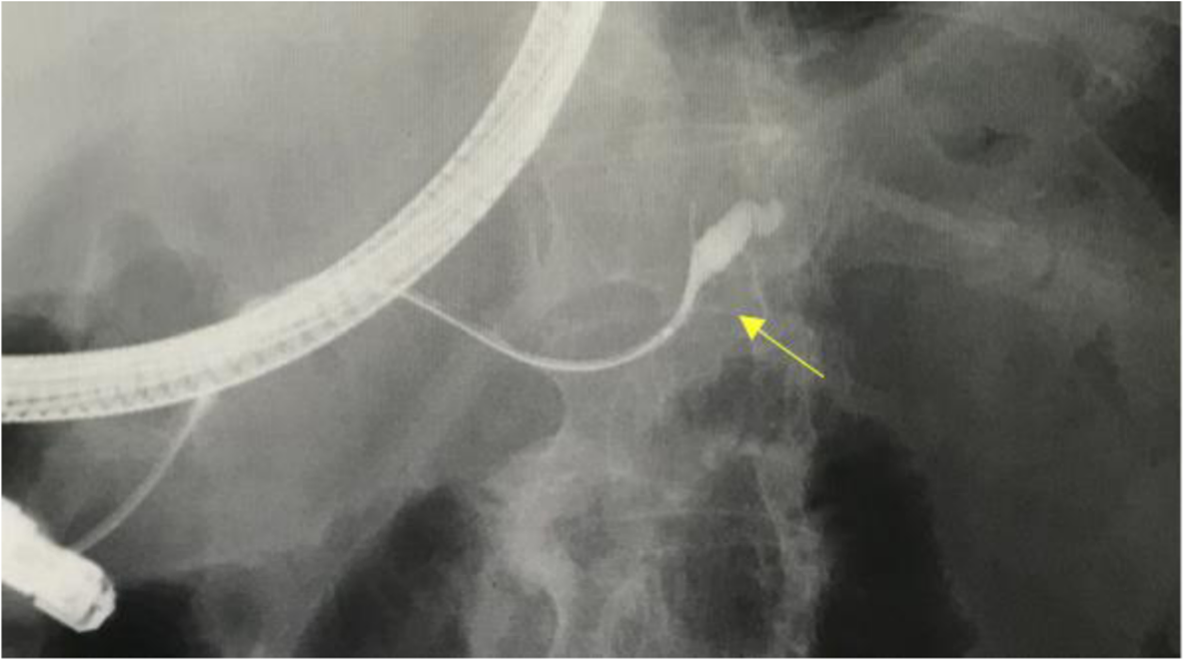
Endoscopic retrograde pancreatography revealed that the main pancreatic duct was stenotic without any connection to the cystic lesion. The main pancreatic duct showed tapered stenosis at the pancreatic body and a slightly dilated main pancreatic duct on the distal side

**Fig. 3:**
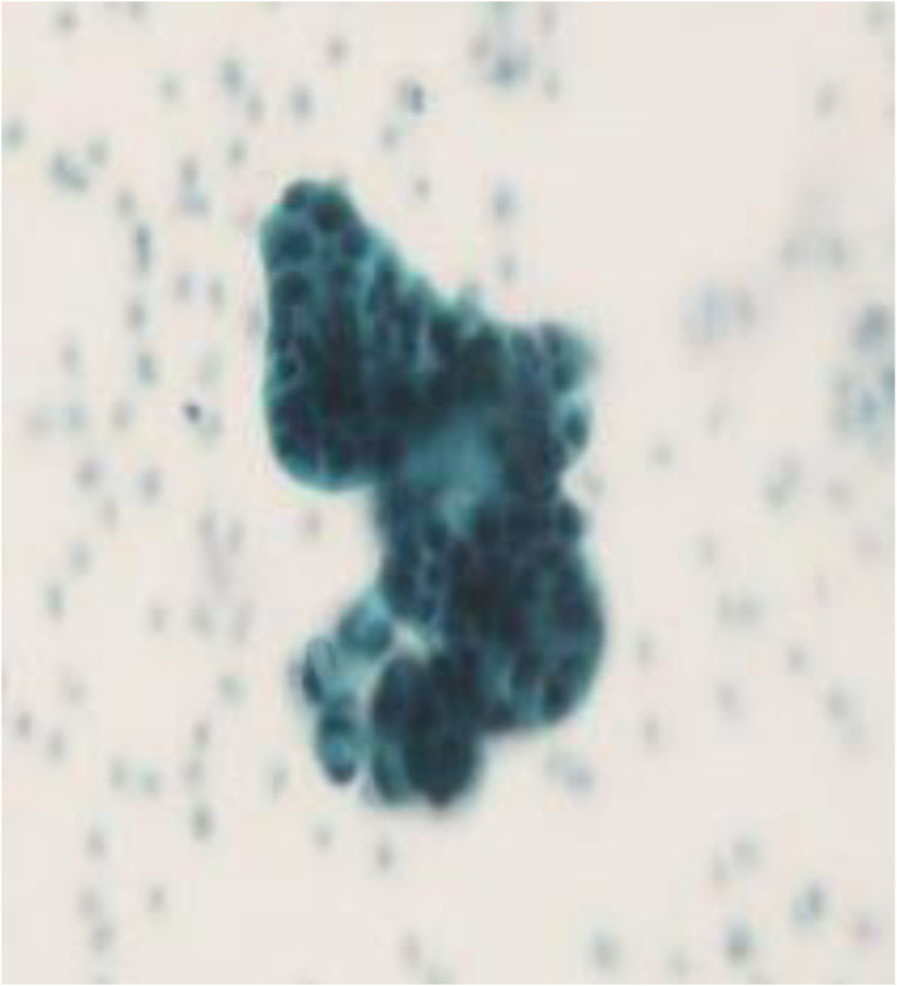
Abdominal magnetic resonance imaging demonstrating T2 high and a fluid-fluid level

**Fig. 4:**
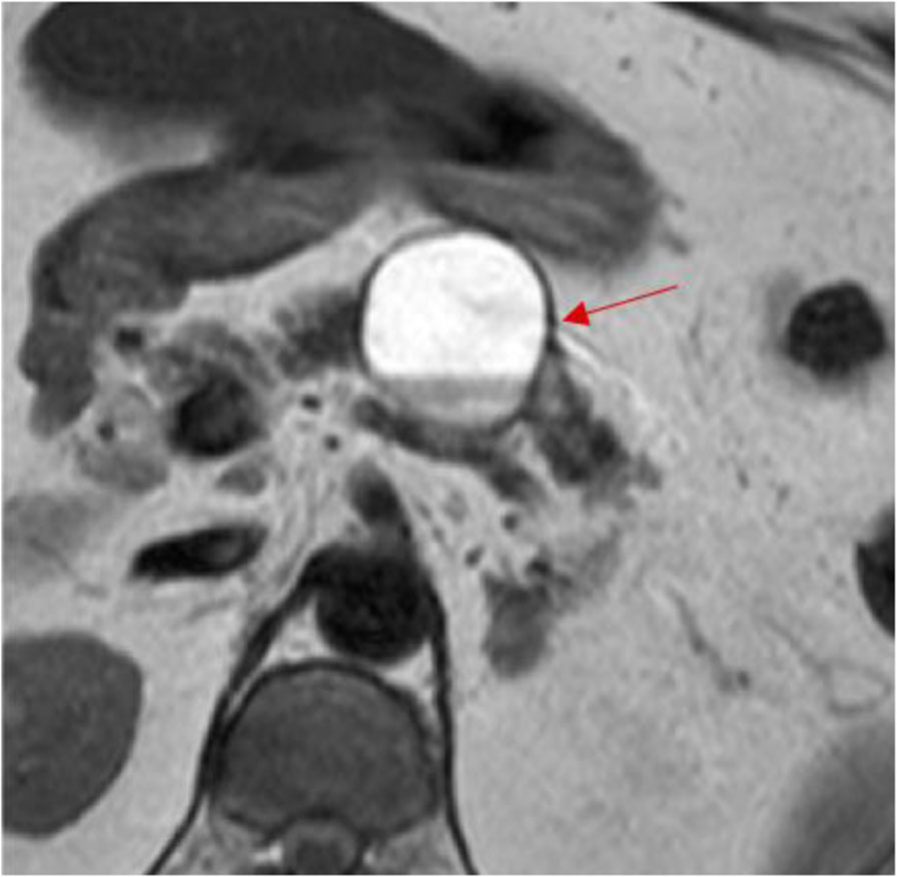
Cytological examination demonstrated class IIIb adenocarcinoma

In 2011, a 66-year-old Japanese woman underwent unenhanced computed tomography (CT) that showed an incidental cystic lesion in the tail of the pancreas measuring 9 mm (Fig. 1a). She did not have any symptoms. After that, she underwent a CT scan every year. Her medical histories were as follows: breast cancer, HCV carrier, hypertension, and type 2 diabetes mellitus. Laboratory tests, including carcinoembryonic antigen (CEA) and carbohydrate antigen 19-9 (CA19-9), were normal. In 2013, the cystic lesion had grown to 14 mm, and the peripheral rim was enhanced on contrast-enhanced CT (CECT) (Fig. 1b). Although the cystic lesion continued to increase gradually, its form did not change. In 2018, the cystic lesion had become 33 mm and contained fluid at the bottom, which was considered clot on unenhanced CT (Fig. 1c). However, endoscopic ultrasound did not reveal a mural nodule lesion. In 2019, the cystic lesion was 40 mm, and the outer rim was thinner on CECT (Fig. 1d). Endoscopic retrograde pancreatography (ERP) showed that the main pancreatic duct was stenotic without any connection to the cystic lesion (Fig. 2). The main pancreatic duct further exhibited tapered stenosis at the pancreatic body and a slightly dilated main pancreatic duct on the distal side. Cytological examination demonstrated class IIIb adenocarcinoma (Fig. 3). The patient also underwent magnetic resonance imaging (MRI), demonstrating T1 low, T2 high, and fluid-fluid levels (Fig. 4). We considered that an intraductal papillary mucinous neoplasm or a mucinous cystic neoplasm was complicated by pancreatic cancer, and we performed laparoscopic distal pancreatectomy. The patient had an uneventful postoperative recovery and left the hospital 12 days later.

**Fig. 5:**
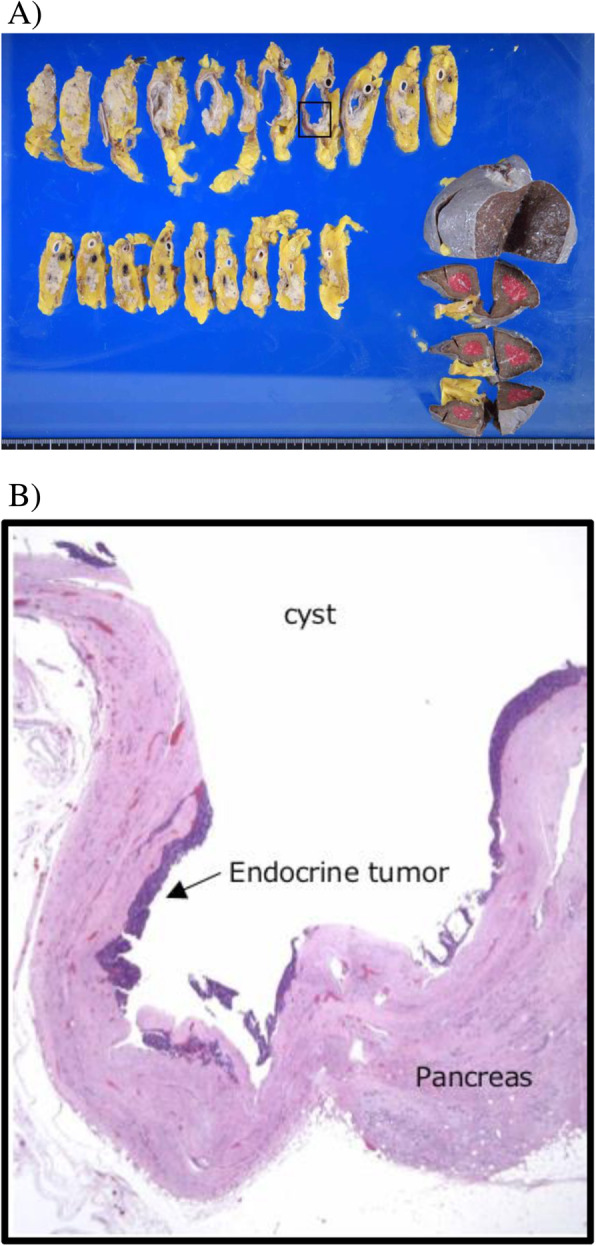
**a** Macroscopic findings demonstrated a cystic lesion measuring 21 × 11 mm in the tail of the pancreas. **b** Histopathology revealed that the wall of the cyst was filled with fibrous connective tissue, with some growth of tumor cells consisting of atypical cells with dense chromosomes on the inner surface (hematoxylin and eosin stain)

**Fig. 6:**
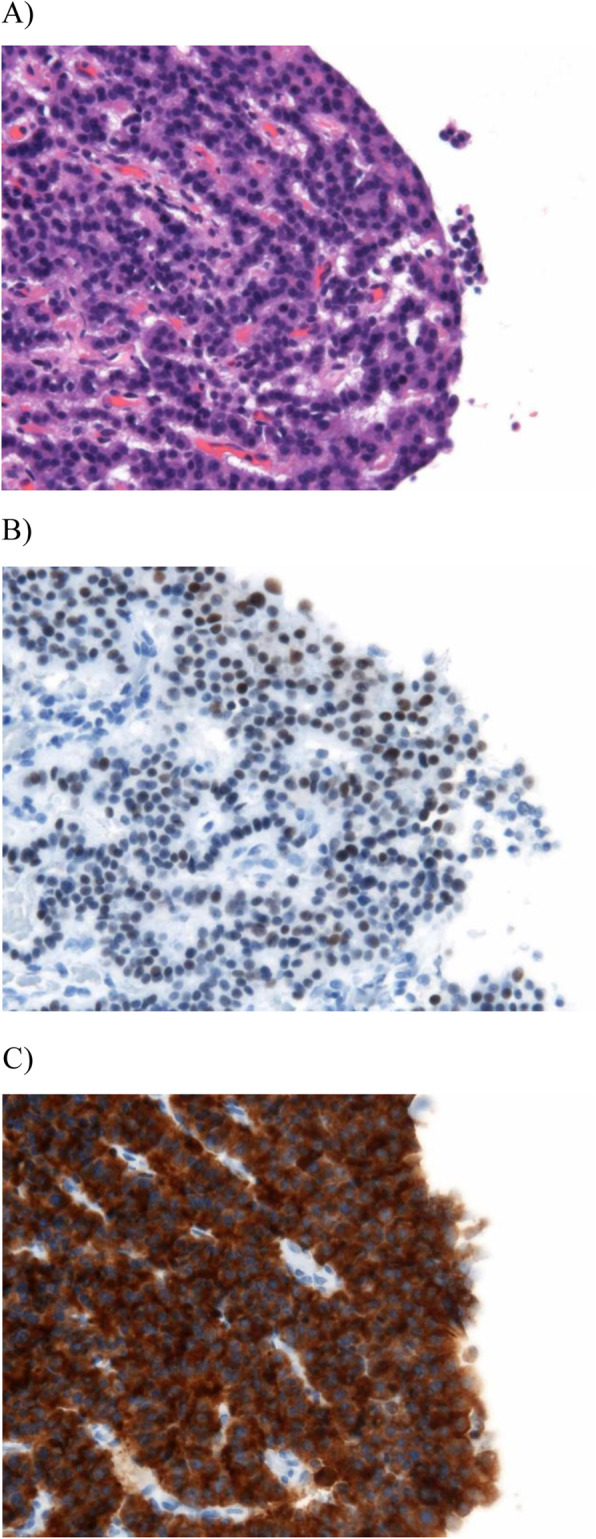
Histopathological findings for the resected pancreas. **a** Tumor cells had ribbon-like hyperplasia and gland-like structures. There were small blood vessels around the cells and no mitotic figures at 10 hpf (hematoxylin and eosin staining). **b** and **c** Tumor cells showed positive results for INSM1 (**b**) and synaptophysin (**c**) by immunohistochemical staining

Histopathology showed a cystic lesion measuring 21 × 11 mm in the tail of the pancreas (Fig. 5a). The content of the cystic lesion was serous fluid without blood. The wall of the cyst was filled with fibrous connective tissue, with some growth of tumor cells consisting of atypical cells with dense chromosomes on the inner surface. There was no necrosis or mucus in the lumen (Fig. 5b). The tumor cells displayed ribbon-like hyperplasia and gland-like structures. There were small blood vessels around the cells and no mitotic figures at 10 hpf (Fig. 6a). Cytological examination showed positive staining for INSM1 and synaptophysin, which are highly specific markers for neuroendocrine tumors (Fig. 6b and c). The Ki-67 index was less than 1%, making this a G1-type tumor. Immunohistochemical markers of insulin and glucagon were negative.

## Discussion

Neuroendocrine tumors account for only 1–2% of all pancreatic neoplasms, and cystic lesions account for less than 10% of all neuroendocrine tumors; thus, cystic neuroendocrine lesions are very rare [1]. However, the increasing number of diagnostic examinations enables more detection of cystic lesions of the pancreas [3]. The mean age at diagnosis is 53 years, and there is no evidence of sex predilection [2]. Overall, cystic PNETs appear to be larger, of a lower grade and present at a lower stage than typical solid PNETs [4, 5]. In addition, incidences of regional lymph node metastases with cystic PNETs are rare compared with typical solid PNETs [6]. Most cystic PNETs are nonfunctional, and the diagnosis is usually made incidentally or secondary to mass-dependent symptoms such as abdominal pain. Except for neuroendocrine microadenomas, all neuroendocrine tumors are considered to have malignant potential and should be considered for surgical resection [6, 7]. However, the diagnosis of cystic PNETs with conventional axial imaging, such as CT and MRI, is extremely difficult [8]. In our case, the tumor increased gradually and the cytology for the specimen obtained by ERP-demonstrated indicated class IIIb adenocarcinoma; therefore, we were not able to achieve a preoperative diagnosis of cystic PNET.

With regard to how preoperative ERP cytology showed class IIIb adenocarcinoma, it was presumed that we had taken some PNET cells as specimens that had fallen into the pancreatic duct from the cystic lesion. In addition, these cells were suspected to be adenocarcinoma on cytology. We have determined that the release of some PNET cells from the cystic lesion may have been caused by the guidewire technique or a very small connection that had not been detected by some diagnostic examinations.

We also considered the mechanism of cystic degeneration. There are two main hypotheses regarding the presence of cystic components in PNETs. Some investigators speculate that the fibrous capsules of PNETs hinder blood supply to the lesion, leading to necrosis and subsequent cystic degeneration [9, 10]; others postulate that hemorrhage may occur in PNETs, which are highly vascular, and that cystic degeneration is a consequence of such hemorrhage [11, 12]. Regardless, the precise mechanisms have not been fully elucidated [5].

In our case, the tumor presented as a cystic lesion when it was first detected in 2011, and histopathology showed some growth of tumor cells on the inner surface of the wall of the cyst, with no necrosis or mucus in the lumen. Consequently, it was presumed that the mechanism of cystic degeneration in our case was not necrosis but intralesional hemorrhage before it was detected.

Nevertheless, we also considered another hypothesis: this lesion had occurred as a small cyst that increased as it was. Indeed, it was detected as a very small cystic lesion that increased without a change in form. These findings suggest that our patient’s PNET developed as a cyst that did not degenerate.

We were able to observe the progress of our case in detail because we had followed up for 8 years.

## Conclusion

Although a cystic change of PNET is generally caused by ischemia or necrosis inside the tumor, some PNETs may occur as a small cyst and increase without changing form.

## Data Availability

All data regarding this paper are included in this published article.

## Abbreviations

PNET: Pancreatic neuroendocrine tumor
CT: Computed tomography
ERP: Endoscopic retrograde pancreatography
CEA: Carcinoembryonic antigen
CA19-9: Carbohydrate antigen 19-9
CECT: Contrast-enhanced CT
MRI: Magnetic resonance imaging
